# User satisfaction in child and adolescent mental health service: Comparison of background, clinical and service predictors for adolescent and parent satisfaction

**DOI:** 10.1111/hex.13861

**Published:** 2023-08-31

**Authors:** Yngvild Arnesen, Kjersti R. Lillevoll, Børge Mathiassen

**Affiliations:** ^1^ Department of Child and Adolescent Psychiatry, Division of Child and Adolescent Health University Hospital of North Norway Tromsø Norway; ^2^ Research Group for Clinical Psychology, Department of Psychology, Faculty of Health Sciences, UiT The Arctic University of Norway Tromsø Norway

**Keywords:** adolescents, child and adolescent mental health services, parents, predictors, user satisfaction

## Abstract

**Background and Objective:**

To improve quality, child and adolescent mental health services (CAMHS) are expected to quantify families' views on healthcare with user satisfaction measures. As little is known about what influences satisfaction in CAMHS, this study aimed to examine predictors of adolescents' and parents' user satisfaction.

**Methods:**

Data from 231 adolescents and 495 parents in treatment at an outpatient clinic who returned a user satisfaction measure, the Experience of Service Questionnaire (ESQ), was analyzed. Registry data on background, clinical and service characteristics were predictors for the ESQ factors general satisfaction, satisfaction with care and satisfaction with environment.

**Results:**

In regression models, satisfaction with care for adolescents (*r* ^2^ = .12) was significant and was predicted by low parent‐self‐reported mental health burden and low clinician‐rated overall symptom burden at intake. For parents, regression models for general satisfaction (*r* ^*2*^ = .07), satisfaction with care (*r* ^*2*^ = .06) and satisfaction with environment (*r* ^*2*^ = .08) were significant. Parents general satisfaction was predicted by higher levels of hyperactivity, less family stress and longer travelling distances to the service. Satisfaction with care for parents was predicted by higher levels of hyperactivity at intake and longer travelling distances. Satisfaction with environment for parents was more likely if the adolescents was a boy, with low levels of family stress and longer travelling distances.

**Conclusion:**

Predictors for adolescent and parent user satisfaction in CAMHS differ. Hence, to improve quality CAMHS should enhance focus on collaborative practice with parents, and person‐centred care for adolescents with moderate to severe mental health illness.

**Patient or Public Contribution:**

Representatives from the hospitals' youth panel and the non‐governmental organization called The Change Factory have been consulted regarding study design and results.

## 1 INTRODUCTION

Currently, high‐quality child and adolescent mental health services (CAMHS) are expected to involve families in decisions regarding their care.^1^ At CAMHS, families meet multidisciplinary teams specialized in comprehensive assessment, diagnostics and treatment of moderate to severe mental health disorders. Determining what constitutes quality at CAMHS remains a topic of ongoing debate. However, the importance of tracking user satisfaction to facilitate family involvement and bridging families' and clinicians' perspectives on the quality of care is often emphasized.^2^ The lack of an established theoretical framework for investigating user satisfaction in CAMHS leaves a gap in the understanding of the concept.^3^ Thus, for user satisfaction to be a meaningful metric for evaluating CAMHS, there is a call for knowledge of factors that impact user satisfaction.

Despite the growing popularity of user satisfaction, still few CAMHS routinely track it,^4^ and the literature on factors related to user satisfaction is ambiguous.^5^, ^6^ To date, most studies have focused on parents, leaving a gap in the available literature regarding the perspectives of adolescents.^5^, ^7^ Furthermore, methodological issues such as lack of psychometric valid user satisfaction measures and low response rates persist.^3^, ^5^, ^8^ Given the discrepancy between adolescents' and parents' attitudes toward CAMHS,^8^, ^9^ this research gap hinders any definitive conclusions, particularly concerning evaluating critical elements of service quality from the perspectives of adolescents.

Considering the available evidence on background variables, some studies find adolescents' gender do not affect responses to satisfaction measures.^3^, ^8^, ^10^, ^11^ Nonetheless, some researchers find boys,^12^ or parents of boys,^13^ report higher satisfaction, while one study found girls reported higher satisfaction with services.^5^ Regarding the satisfaction and age of the adolescents, Bjørngaard et al.^14^ found parents of younger children reported the highest satisfaction. Along the same lines, Stüntzner‐Gibson et al.^15^ reported younger teenagers were more satisfied than older teenagers, but more recently, McNicholas et al.^9^ found being a late teen best‐predicted satisfaction. Further, some evidence shows the weak influence of socioeconomic background variables on user satisfaction for both adolescents and parents,^5^ while more recent studies indicate parental ethnicity may influence satisfaction.^16^, ^17^ The predictive power of other potential background variables affecting the dynamics of families like stress, parental mental health or characteristics of the adolescents needs further exploration.^16^, ^18^

Treatment satisfaction and symptom relief are separate constructs,^8^ and the association between how the two relate is uncertain.^3^, ^4^, ^19^ Parallel to healthcare in general,^20^ previous studies show adolescents with more severe diagnosis report lower levels of satisfaction with CAMHS.^11^, ^21^, ^22^ Another reoccurring finding has been externalizing problems as a predictor for dissatisfaction.^14^, ^21^, ^22^ Notable, Urben et al.^23^ found adolescents with low emotional symptom burden at intake were more satisfied. Interestingly, in recent studies, Kapp et al.^5^ found no associations between the severity of the disorder and satisfaction, and McNicholas et al.^9^ concluded that those with no diagnosable mental health conditions were least likely to be satisfied with CAMHS.

While the literature reveals inconsistencies regarding evidence for background and clinical variables, predictors relating to the organization of services have reoccurred. Having quick access to services^5^, ^14^, ^24^ and the opportunity to stay in services longer^14^, ^25^ with frequent,^13^ structured and goal‐oriented contact^9^, ^26^ benefits satisfaction. Services providing user‐friendly, easy‐access information to minimize families' queries about what to expect when visiting CAMHS demonstrably lead to more satisfied families.^27^, ^28^, ^29^ Families seen at services where they get included in treatment planning, get a choice in deciding the frequency of sessions and are ensured by the approach to treatment at intake report high levels of satisfaction.^5^ Two studies, in addition, suggest that living near the service is profitable,^9^, ^13^ suggesting CAMHS services should not cover large geographical areas. In a review of the literature on satisfaction in CAMHS, Biering^7^ also highlights the importance of the environment and organization of services. Yet intuitively acceptable, these findings must be regarded with some caution. McNicholas et al.^9^ did not report any association between satisfaction and waiting time, and a study by Urben et al.^23^ did not find any association between the duration of treatment and satisfaction.

Given the ambiguous results of previous studies, expanding the understanding of user satisfaction in CAMHS is essential. Multiple reasons underscore the necessity of this endeavour, including the need to engage families effectively during the care pathway, bridging the perspectives of families and clinicians, improving treatment outcomes, assessing and improving quality and promoting accountability within CAMHS. Previous research has investigated a limited set of predictors, leading to an inadequate understanding of the user satisfaction construct. To address these gaps, the primary aim of this study was to augment the existing knowledge by examining possible factors influencing user satisfaction among adolescents and parents in CAMHS, with a specific focus on variables identifiable during the initial intake at services. More specifically, we aimed to explore which background, clinical and service factors, as assessed during intake, could predict individual variation in user satisfaction. User satisfaction was quantified utilizing the Experience of Service Questionnaire (ESQ),^30^ which encompasses a general factor for satisfaction as well as subordinate factors for satisfaction with the care and satisfaction with environment.^31^ To our knowledge, no previous study has explored predictors of adolescent and parent general satisfaction, satisfaction with care and satisfaction with the environment in routine clinical practice.

## 2 METHODS

### 2.1 Participants

**Figure 1:**
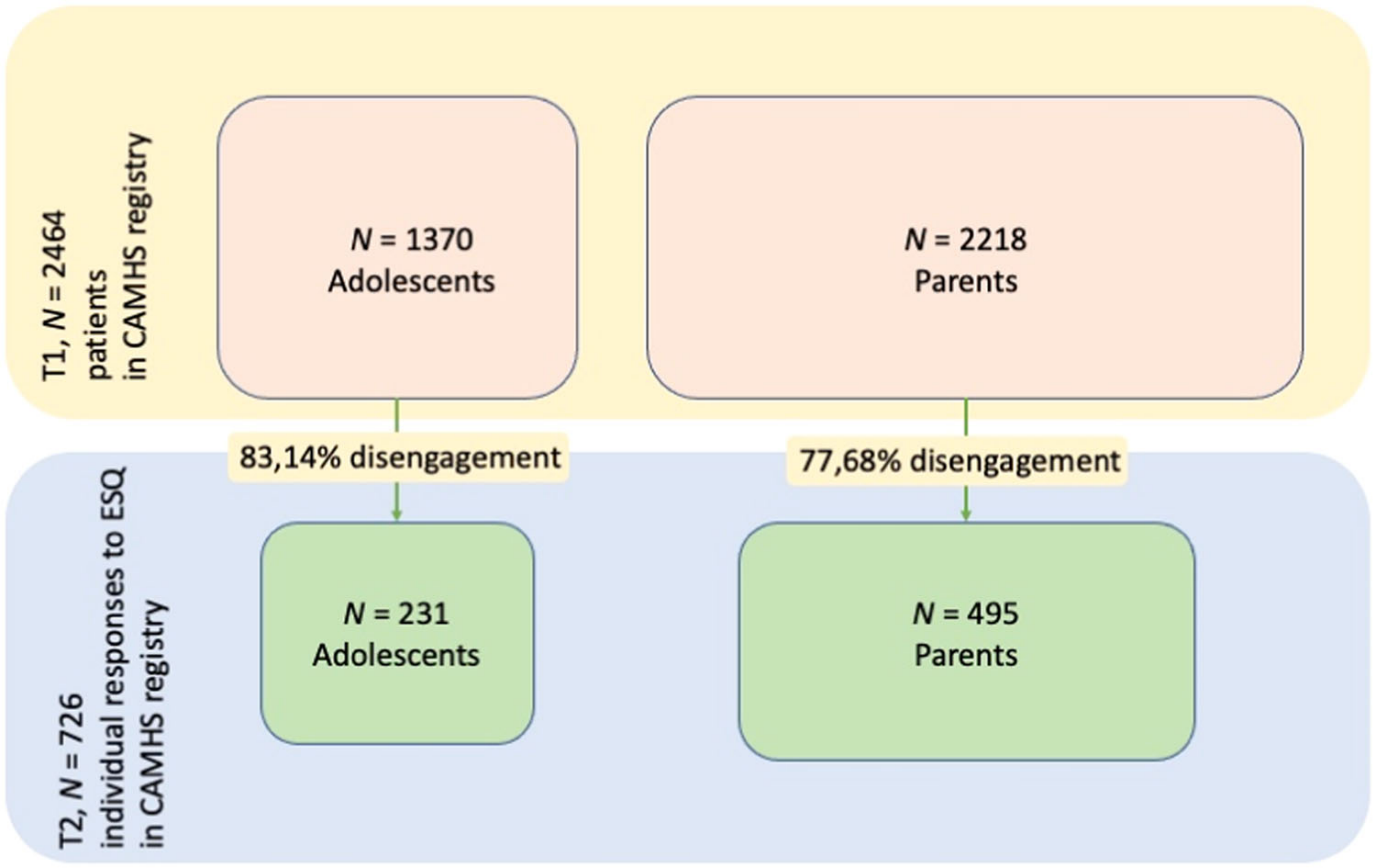
Data inclusion flow chart. CAMHS, child and adolescent mental health services.

A quality registry data set from the University Hospital Trust of Northern Norway, including data on patients receiving outpatient treatment at CAMHS, was utilized. In Norway, children and adolescents are referred to CAMHS by general practitioners, other specialists at the hospital trust, community psychologists or social services. All patients eligible for CAMHS between the 1 December 2013 and the 31 December 2020 were included in the registry. The registry holds data from the electronic patient record and routine outcome measures from adolescents, parents, and clinicians. During the inclusion period, 2429 children and adolescents were referred to the service and granted patient rights. To be eligible for this study, adolescents or parents had to complete the corresponding version of the ESQ 6 months after intake (T2). Adolescents were invited to complete the ESQ from the age of 11, while parents completed the ESQ regardless of the age of their child/adolescent. The registry included ESQ responses from 726 individuals, with more parents (*n* = 495) than adolescents (*n* = 231). A power analysis (*α* = .05, power = 0.80) was conducted before the study, indicating a minimum sample size of 131 participants to detect a medium effect size (*f* ^2^ = 0.15) in a regression analysis with 13 predictors. For further details regarding participants and disengagement, see Figure 1 for the data inclusion flow chart.

### 2.2 Data collection procedures

The routine outcomes measures in the quality registry were collected at intake (T1) and follow‐up 6 months later (T2) from the adolescents, parents, and clinicians, in accordance with the child outcome research consortium snapshot protocol (https://www.corc.uk.net/resource-hub/sending-data-to-corc/).^32^, ^33^ Data were collected digitally from adolescents and parents through the youth‐in‐mind‐portal (https://youthinmind.com/), which includes the Development and Well‐being Assessment (DAWBA),^34^ the Strength and Feelings Questionnaire (SDQ)^35^ at T1, and: the ESQ was collected at T2. Before meeting for the initial session (T1) and evaluation session (T2) at CAMHS, families received a detailed letter describing the use of routine outcome measures, the log‐in procedures at the youth‐in‐mind‐portal and a separate sealed envelope for each family member with their personal log‐in‐code. The letter emphasized completing the measures is voluntary and offered a phone support service for questions. Data from clinicians were collected by paper and entered manually into the registry by a secretary at the clinic. If measures were not completed, the secretary reminded families once by phone, and clinicians via the electronic patient record system. The local data protection officer at the hospital trust, who acts on behalf of the Norwegian data protection authority, approved the study. Written consent was not required as data were collected routinely at the clinic for quality assurance, procedures for safe storage were followed, and only deidentified data was retrieved from the quality registry for secondary analysis for this study.

### 2.3 Measures

#### 2.3.1 Satisfaction

The ESQ has 12 items rated on a 3‐point Likert scale (not true, partly true, certainly true). Items answered ‘don't know’, and the three open‐ended questions inviting free‐text responses were not included in this study. The factor general satisfaction includes all items and has a range of 0–36, while the factor satisfaction with care (items: 1–7, 11 and 12) has a range of 0–27, and the factor satisfaction with the environment (items: 8–10) has a range of 0–9. The ESQ is freely available, used internationally and in evaluations recommended to supplement measures of clinical change.^31^, ^36^

#### 2.3.2 Background characteristics

*Family stress*: Parents' reports of perceived stress were measured using the family stress scale, which is a part of the DAWBA.^34^ The 13 items comprising the scale are rated on a 3‐point scale (0 = no, 2 = a lot), yielding a maximum of 26. The scale includes questions on household financial conditions, unemployment, housing, neighbourhood, tensions with partner or ex‐partner, illness, gambling‐, alcohol‐ or drug‐misuse. High scores indicate more ongoing stress on the family. Validation of the family stress scale remains. *Parent mental health*: Parents self‐reported psychological well‐being and distress during the last month on the Everyday Feeling Questionnaire,^37^ also part of the DAWBA. Ten items regarding levels of well‐being and distress for parents are rated on a 5‐point scale (0 = no, 4 = a lot), with a maximum of 40 (mean = 11.59, SD = 5.05).^37^, ^38^ Cronbach's *α* Everyday Feeling Questionnaire is reported between .87 and .90.^37^, ^38^, ^39^ *Peer problems*: Both adolescents and parent‐reported levels of peer problems were measured with the SDQ subscale peer problems, including queries about bullying, preferring to play alone and so forth.^35^ Each of the SDQ subscales includes five items scored on a 3‐point scale (from 0 = not true, to 2 = true) with a maximum of 10. The SDQ has proven good psychometric properties for the Norwegian version, and the peer problems subscale have shown Cronbach's *α* of .44–.64 for adolescents and .43–.75 for parents.^40^, ^41^ *SDQ prosocial skills*: Adolescents and parent‐reported levels of prosocial skills were measured with the SDQ subscale prosocial skills describing if the adolescents are typically kind to younger children, helps out and so forth. The prosocial skills subscale has shown Cronbach's *α* of .62–.66 for adolescents, and between .62–.80 for parents.^40^, ^41^

#### 2.3.3 Clinical characteristics

*Children's Global Assessment Scale (CGAS)*. The severity of adolescents' daily psychosocial function was measured by the CGAS.^42^ Clinicians rate functioning from 1 (needs constant supervision) to 100 (superior function in all areas), where higher scores indicate higher daily functioning. The reliability and validity of the CGAS is well documented. CGAS above 70 at intake is usually considered a non‐case at CAMHS (mean = 55.9, SD = 6.9).^43^ Inter‐rater reliability among experienced CGAS‐raters is good.^44^, ^45^

*The Health of the Nation Outcome Scales of Children and Adolescents (HoNOSCA)* ^46^ is a rating scale covering mental health problems and symptoms used by clinicians to indicate the overall symptom burden and clinical severity. In this study, 13 of 15 subscales scored between 0 (no problem) and 4 (severe to very severe problem), were summed to a total score (range: 0–52). There is no clinical cut‐off for total HoNOSCA, although ratings in outpatient clinics typically show a mean of 12.0 (SD = 4.6)^47^ the Norwegian version has good psychometric properties.^48^ *Strengths and Difficulties Questionnaire (SDQ)*.^35^ The subscales for emotional problems (e.g., worries, unhappy), conduct problems (e.g., fights, lies) and hyperactivity (e.g., restless, distractable, inattentive) were chosen as they cover most of the symptom burden in a clinical population. The psychometric properties of the Norwegian version of the SDQ is well documented.^40^, ^41^ For the subscale emotional problems Cronbach's *α* range from .61–.73, for conduct problems Cronbach's *α* range from .38–.59, and for hyperactivity Cronbach's *α* range from .53–.76 for adolescents.^40^ For parents Cronbach's *α* range from .45–.70 for emotional problems, .45–.75 for conduct problems and .75–.80 for hyperactivity.^41^

#### 2.3.4 Service characteristics

*Waiting time* was recorded in days from referral to the first physical meeting between family and clinician at CAMHS. Waiting time was imported from the electronic patient journal to the registry by the secretary. *Travel distance* was imported from the electronic patient journal. Distance to service was dummy coded as 0 if families lived within the municipality of the CAMHS, and 1 if the family lived outside the municipality of the CAMHS (typically having above 1‐h travelling distance to the service).

### 2.4 Statistical analysis

Data were analyzed using SPSS statistics 27. As satisfaction scores are typically skewed, nonparametric tests were run to compare adolescents and parents. Pearson correlation was used to examine the association between the dependent variables and predictors. Regression analyses were conducted separately for adolescents and parents, with the ESQ factors general satisfaction, satisfaction with care and satisfaction with environment as outcome variables. Three models were tested for each group. A Bonferroni correction (*α* = .05/3) was conducted post hoc for each group to control for family‐wise error rates.

The multi‐informant data set revealed missing items for the study variables ranging from 0% to 31.4% (for details see Table 1). Missing values were missing at random and replaced by multiple imputations (*n* = 5), created with the fully conditional specification method, including all available variables for each sample. The imputed datasets were pooled together to form one complete data set for each sample, enabling subsequent analysis on the full set of variables. After removing five outliers in the adolescents' sample, multicollinearity among predictors was not a concern as analysis revealed variance inflation factors were well below 2.5 (range: 1.019–1.922 in both samples), and tolerance above 0.1 for all predictors. As expected, the *q*–*q* plot of residuals revealed skewness for all ESQ factors in both samples.

**Table 1:** Descriptive statistics imputed samples presenting problems and study variables.

	Adolescents (*n* = 231)	Missing (%)	Parents (*n* = 495)	Missing (%)
Age of adolescent (mean/SD)	14.06 (1.91)		11.16 (3.43)	
Gender (*n*/%)				
Girls	154/66.70		230 (46.50)	
Presenting problems at intake				
SDQ total score (mean/SD)	16.58 (5.39)		16.16 (6.38)	
DAWBA any disorder (*n*/%)	157/67.40		339/68.80	
DAWBA emotional disorder (*n*/%)	134/57.50		196/39.60	
DAWBA conduct disorder (*n*/%)	44/18.90		154/31.10	
DAWBA hyperactive disorder (*n*/%)	33/14.20		73/14.70	
Study variables				
Family stress^a^ (mean/SD)	2.23 (2.13)	17.4	2.31 (2.19)	9.1
Parent mental health^b^ (mean/SD)	12.60 (4.10)	17.8	13.25 (4.80)	9.3
Peer problems^c^ (mean/SD)	3.32 (2.12)	2.1	3.29 (2.37)	2.4
Prosocial skills^c^ (mean/SD)	7.69 (1.84)	2.1	7.12 (2.21)	2.4
Daily function^d^ (mean/SD)	54.32 (8.55)	13.1	54.24 (7.37)	14.5
Overall symptoms^e^ (mean/SD)	12.98 (4.74)	31.4	12.21 (4.41)	26.5
Emotional symptoms^c^ (mean/SD)	6.01 (2.61)	2.1	4.80 (2.68)	2.4
Conduct problems^c^	2.29 (1.65)	2.1	2.96 (2.10)	2.4
Hyperactivity^c^	4.97 (2.38)	2.1	5.12 (2.80)	2.4
Waiting time (days)	54.5 (27.86)	5.9	58.97 (27.15)	5.7
Travelling distance^f^ (*n*/%)				
City centre	162/70.1	5.9	393/179.40	5.7
Rural	69/29.90	5.9	102/20.60	5.7

Abbreviations: CGAS, Children's Global Assessment Scale; DAWBA, Development and Well‐being Assessment; HoNOSCA, The Health of the Nation Outcome Scales of Children and Adolescents; SDQ, Strength and Feelings Questionnaire.

^a^ Family stress scale, total score parent.

^b^ Everyday Feeling Questionnaire, total score parent.

^c^ Separate SDQ ratings satisfaction for adolescents and parents.

^d^ CGAS intake score.

^e^ HoNOSCA intake score.

^f^ City centre <1‐h travelling distance = 0, rural >1‐h travelling distance = 1.

## 3 RESULTS

In the total registry sample (*n* = 2429) *M*_age_ = 11.91, SD_age_ = 4.24, range: 0–19 years; girls 50.5%. Disengagement between T1 and T2 was 83.14% for adolescents and 77.68% for parents. Details of the adolescents (*n* = 231) and parent (*n* = 495) study samples are found in Table 1. In the study samples, nearly 70% of the adolescents had a diagnosable mental health disorder at intake. The mean total reported difficulty score at intake (SDQ total score) resembled other clinical samples in Norway.^40^, ^41^ Emotional disorders were more common in the adolescent sample, while conduct disorders were more frequent for adolescents in the parent sample. Hyperactive disorders were equally common in the adolescents and parent samples.

Satisfaction scores were highly skewed, especially for the parent sample who were significantly more satisfied than adolescents on all satisfaction scales: general satisfaction (Wilcoxon‐signed ranks test, *z* = −3.43, *p* = .001), satisfaction with care (Wilcoxon‐signed ranks test, *z* = −2.92, *p* = .003), and satisfaction with environment (Wilcoxon‐signed ranks test, *z* = −2.30, *p* = .021). Further details are shown in Table 2.

**Table 2:** Level of satisfaction.

	Adolescents			Parents		
	Mean	SD	Maximum score (*n*/%)	Mean	SD	Maximum score (*n*/%)
General satisfaction	29.39	7.33	42/18	31.68	5.97	126/25.5
Satisfaction with care	21.97	6.06	69/29.6	23.90	4.87	218/44
Satisfaction with environment	7.42	1.76	82/35.2	7.78	1.63	222/44.8

### 3.1 Bivariate analysis

Bivariate analysis for adolescents (Table 3) revealed that general satisfaction and satisfaction with care were negatively correlated with parent‐self‐reported mental health (general satisfaction = −.16*, satisfaction with care = −.18**), clinician‐rated overall symptoms (general satisfaction = −.15*, satisfaction with care = −.17*), adolescents‐reported conduct problems (general satisfaction = −.13*, satisfaction with care = −.15*) and hyperactivity problems (general satisfaction = −.16*, satisfaction with care = −.18**). Adolescents' self‐reported prosocial skills were positively correlated with both general satisfaction (.15*) and satisfaction with care (.16*). No significant correlations were found between satisfaction with the environment for adolescents and the predictor variables.

**Table 3:** Correlations between dependent variables and predictors for adolescents.

	1.	2.	3.	4.	5.	6.	7.	8.	9.	10.	11.	12.	13.	14.	15.
Dependent variables															
1. General satisfaction															
2. Satisfaction with care	.98**														
3. Satisfaction with environment	.78**	.66**													
Predictors															
4. Age	−.01	−.04	.07												
5. Gender a	.00	−.01	.02	.19**											
6. Family stress^b^	.01	−.02	.10	.07	−.03										
7. Parent mental health^c^	−.16*	−.18**	−.06	.00	−.01	.44**									
8. Peer problems^d^	−.05	−.02	−.11	.00	−.01	.06	.03								
9. Prosocial skills^d^	.15*	.16*	.05	−.02	.15*	−.14*	−.07	−.14*							
10. Daily function^e^	.07	.06	.08	−.01	.07	.08	.01	−.19**	.14*						
11. Overall symptoms^f^	−.15*	−.17*	−.05	.16*	.06	.01	.02	.19**	−.20**	−.62**					
12. Emotional symptoms^d^	.03	.02	.05	.27**	.37**	.03	.00	.25**	.05	−.15*	.19**				
13. Conduct problems^d^	−.13*	−.15*	−.02	−.04	−.05	.13*	.08	.12	−.27**	−.08	.28**	.01			
14. Hyperactivity^d^	−.16*	−.18**	−.07	.19**	−.08	.09	.05	−.08	−.13*	−.03	.16*	.13*	.53**		
15. Waiting time (days)	.04	.06	−.03	.04	−.16*	.06	.09	.02	−.08	.16*	.03	−.06	−.01	−.01	
16. Travelling distance^g^	.00	.03	−.08	.13*	.10	−.13*	.02	.00	−.06	−.05	.05	.10	.09	.11	.22**

^a^ Boy 1, girl 2.

^b^ Family stress scale, total score parent.

^c^ Everyday Feeling Questionnaire, total score parent.

^d^ Strength and Feelings Questionnaire ratings youth.

^e^ Children's Global Assessment Scale intake score.

^f^ The Health of the Nation Outcome Scales of Children and Adolescents intake score.

^g^ <1 h travelling distance = 0, >1 h travelling distance = 1.

* *p* < .05

** *p* < .01 (two‐tailed test).

In the corresponding parent bivariate analysis (Table 4), significant negative correlations between general satisfaction, satisfaction with care and satisfaction with environment and age (general satisfaction = −.13**, satisfaction with care = −.11*, satisfaction with environment = −.14**), family stress (general satisfaction = −.11*, satisfaction with care = −.09*, satisfaction with environment = −.11*), peer problems (general satisfaction = −.12*, satisfaction with care = −.09*, satisfaction with environment = −.14**), and clinician‐rated overall symptoms (general satisfaction = −.10*, satisfaction with care = −.09*, satisfaction with environment = −.09*) were evident. Parent‐reported prosocial skills were positively correlated with general satisfaction (.10*) and satisfaction with environment (.12**). In addition, general satisfaction (−.10*) and satisfaction with environment (−.12**) were negatively correlated with gender, indicating that parents of boys were more likely to be satisfied. Regarding child/adolescent mental health, satisfaction with environment correlated negatively with emotional problems (−.09*), while general satisfaction (.10*) and satisfaction with care (.10*) correlated positively with hyperactivity. General satisfaction (.09*) and travelling distance were positively correlated.

**Table 4:** Correlations between dependent variables and predictors for parents.

	1.	2.	3.	4.	5.	6.	7.	8.	9.	10.	11.	12.	13.	14.	15.
Dependent variables															
1. General satisfaction															
2. Satisfaction with care	.98**														
3. Satisfaction with environment	.75**	.58**													
Predictors															
4. Age	−.13**	−.11*	−.14**												
5. Gender^a^	−.10*	−.08	−.12**	.31**											
6. Family stress^b^	−.11*	−.09*	−.11*	.01	−.02										
7. Parent mental health^c^	−.06	−.05	−.07	.02	.01	.44**									
8. Peer problems^d^	−.12*	−.09*	−.14**	.08	.01	.17**	.15**								
9. Prosocial skills^d^	.10*	.08	.12**	−.02	.06	−.13**	−.14**	−.26**							
10. Daily function^e^	.05	.05	.04	−.05	.04	−.04	−.13**	−.14**	.16**						
11. Overall symptoms^f^	−.10*	−.09*	−.09*	.19**	.03	.01	.07	.25**	−.24**	−.48**					
12. Emotional symptoms^d^	−.06	−.05	−.09*	.07	.22**	.15**	.17**	.33**	−.12**	−.14**	.20**				
13. Conduct problems^d^	−.03	−.02	−.06	−.24**	−.14**	.13**	.17**	.22**	−.52**	−.11*	.22**	.08			
14. Hyperactivity^d^	.10*	.10*	.06	−.30**	−.19**	.05	.01	.13**	−.26**	−.06	.16**	−.02	.54**		
15. Waiting time (days)	.01	.03	−.06	−.12**	−.08	−.05	.00	.03	−.14**	.14**	−.03	.02	.14**	.09*	
16. Travelling distance^g^	.09*	.09	.07	.09	.13**	.02	−.08	.06	−.04	.00	−.05	−.02	.03	.04	.08

^a^ Boy 1, girl 2.

^b^ Family stress scale, total score parent.

^c^ Everyday Feeling Questionnaire, total score parent.

^d^ Strength and Feelings Questionnaire ratings youth.

^e^ Children's Global Assessment Scale intake score.

^f^ The Health of the Nation Outcome Scales of Children and Adolescents intake score.

^g^ <1 h travelling distance = 0, >1 h travelling distance = 1.

* *p* < .05

** *p* < .01 (two‐tailed test).

### 3.2 Regression analysis

Tables 5 and 6 report results from the regression analysis for adolescents and parents on all three dependent variables.

**Table 5:** Regression model for adolescent general satisfaction, satisfaction with care and satisfaction with the environment.

	Adolescent		
	*β*		
	GS	SWC	SWE
Background			
Age	.05	**.03**	.11
Gender^a^	−.03	**−.03**	−.08
Family stress^b^	.13	**.12**	.15
Parent mental health^c^	−.21*	**−.22** **	−.11
Peer problem^d^	−.07	**−.03**	−.17*
Prosocial^d^	.11	**.12**	.03
Clinical characteristics			
Daily function^e^	−.07	**−.10**	.06
Overall symptoms^f^	−.14	**−.18** *	.02
Emotional^d^	.08	**.07**	.10
Conduct^d^	.03	**.01**	.08
Hyperactivity^d^	−.17*	**−.16**	−.17*
Service characteristics			
Waiting time (days)	.07	**.09**	−.02
Proximity to service^g^	.03	**.05**	−.05

*Note*: Bold values indicates a significant regression model with Bonferroni correction.

Abbreviations: GS, general satisfaction; SWC, satisfaction with care; SWE, satisfaction with environment.

^a^ Boy 1, girl 2.

^b^ Family Stress Scale, total score parent.

^c^ Everyday Feeling Questionnaire, total score parent.

^d^ Strength and Feelings Questionnaire ratings according to respondent.

^e^ Children's Global Assessment Scale intake score.

^f^ The Health of the Nation Outcome Scales of Children and Adolescents intake score.

^g^ <1 h traveling distance = 0, >1 h traveling distance = 1.

* *p* < .05

** p < .01.

**Table 6:** Regression model for parent general satisfaction, satisfaction with care and satisfaction with environment.

	Parent		
	*β*		
	GS	SWC	SWE
Background			
Age	**−.07**	**−.05**	**−.09**
Gender^a^	**−.09**	**−.07**	**−.12** *
Family stress^b^	**−.10** *	**−.09**	**−.10** *
Parent mental health^c^	**.02**	**.02**	**.02**
Peer problem^d^	**−.07**	**−.06**	**−.08**
Prosocial^d^	**.06**	**.06**	**.05**
Clinical characteristics			
Daily function^e^	**.00**	**.00**	**.00**
Overall symptoms^f^	**−.07**	**−.07**	**−.04**
Emotional^d^	**.02**	**.03**	**.00**
Conduct^d^	**−.06**	**−.05**	**−.06**
Hyperactivity^d^	**.13** *	**.13** *	**.08**
Service characteristics			
Waiting time (days)	**−.02**	**.01**	**.08**
Proximity to service^g^	**.11** *	**.10** *	**.10** *

*Note*: Bold values indicates a significant regression model with Bonferroni correction.

Abbreviations: GS, general satisfaction; SWC, satisfaction with care; SWE, satisfaction with environment.

^a^ Boy 1, girl 2.

^b^ Family Stress Scale, total score parent.

^c^ Everyday Feeling Questionnaire, total score parent.

^d^ Strength and Feelings Questionnaire ratings according to respondent.

^e^ Children's Global Assessment Scale intake score.

^f^ The Health of the Nation Outcome Scales of Children and Adolescents intake score.

^g^ <1 h travelling distance = 0, >1 h travelling distance = 1.

* *p* < .05.

#### 3.2.1 Predictors of adolescent satisfaction

The regression model for adolescents' *satisfaction with care* was significant with the Bonferroni corrected *p*‐value of .0167 (*f* [13, 222] = 2.210, *p* < .010, *r* ^2^ = .12), accounting for 12% of the variance. Low scores by parents on the Everyday Feeling Questionnaire (*β* = −.22, *p* < .01) and lower clinician‐rated overall symptom burden (HoNOSCA) at intake (*β* = −.18, *p* < .05) were significant predictors of adolescents' satisfaction with care. The regression model for adolescents' *general satisfaction* (*f* [13, 222] = 1.862, *p* < .036, *r* ^2^ = .10) was significant before, but not after the Bonferroni correction. The regression model for *satisfaction with the environment* (*f* [13, 222] = 1.36, *p* < .178, *r* ^2^ = .07) was not significant.

#### 3.2.2 Predictors of parent satisfaction

Parent regression models for all ESQ factors were significant regardless of applying the Bonferroni correction (*p* < .0167). Results showed a significant regression model for parent *general satisfaction* (*f*[13, 478] = 2.790, *p* < .001, *r* ^2^ = 0.07), explaining 7% of the variance in general satisfaction. Significant predictors of general satisfaction reported by parents were less family stress *(β* = −.10, *p* < .05), higher levels of child hyperactivity symptoms at intake *(β* = .13, *p* < .05), and longer travelling distance *(β* = −.11, *p* < .05) to CAMHS. The regression model for parent *satisfaction with care* was significant, *f* [13, 478] = 2.271, *p* < .007, *r* ^2^ = .06), explaining 6% of the variance in satisfaction with care. Parent‐reported hyperactivity symptoms *(β* = −.13, *p* < .05) at intake and longer travelling distances *(β* = −0.11, *p* < .05) to CAMHS were significant predictors. Parent *satisfaction with environment* was significantly explained by the model (*f* [13, 478] = 3.002, *p* < .007, *r* ^2^ = .08), accounting for 8% of the variance. Gender was a significant predictor (*β* = −.12, *p* < .05), indicating parents of boys were more likely to be satisfied. In addition, less perceived family stress (*β* = −.10, *p* < .05), and longer travelling distances (*β* = −.10, *p* < .05) to CAMHS, were significant predictors.

## 4 DISCUSSION

The present study aimed to identify predictors of user satisfaction in CAMHS. By analysing a large sample of routinely collected data, we examined associations between a broad range of background, clinical and service predictors and user satisfaction for adolescents and parents.

The results revealed different factors predicted user satisfaction for adolescents and parents. Our model for adolescent satisfaction with care explained more variance in predictors than the parent models. For adolescents, higher levels of user satisfaction were associated with good parental mental health, and lower levels of clinicians‐rated symptoms at intake. On the other hand, parents reported higher levels of user satisfaction when they perceived less family stress, their child/adolescent had more hyperactivity symptoms, and when they had to travel a longer distance to access CAMHS.

While the models tested in this study explained a substantial amount of variance in predicting user satisfaction compared to some previous models,^8^ they were not as comprehensive as others^3^, ^5^, ^49^ described in the literature. An exception is found for adolescents, where our results regarding satisfaction with care explaining 12% of the variance in predictors resembles findings by Garland, Haine.^3^ Notably, even though an association between parents' socioeconomic status and childhood mental health problems in Norway is evident,^50^ the key cross‐informant effect regarding parent‐reported mental health burden and clinician‐rated symptom burden at intake as predictors of adolescent satisfaction are novel. These findings highlight the importance of addressing parental well‐being and engagement with services in influencing adolescents' experiences at CAMHS. A plausible interpretation for these findings could be these factors contribute to a more supportive and stable environment for adolescents during a vulnerable phase of their upbringing. Parents who have better mental health may be more inclined and able to positively interact with and in line with CAMHS. This may also have a positive impact in adolescents' satisfaction with care. Also, when clinicians rate adolescents with fewer symptoms at intake, this suggest lower severity of their mental health issues, perhaps leading to a more manageable and successful treatment experience. This, combined with the support and understanding from their parents, might contribute to fostering a positive therapeutic alliance and increased satisfaction with care received. If CAMHS focus on addressing parental mental health and rapid reduction of symptom burden during intake, then they can create a more favourable context for adolescents' treatment experiences thus enhancing their satisfaction with CAMHS. Notably, these results also line up with others have also found that adolescents who self‐report lower symptom burden at intake are more satisfied with CAMHS.^23^ In addition, our results are in line with most studies which find gender does not affect adolescent satisfaction.^3^, ^8^, ^10^, ^11^ Neither, the predictive power of service characteristics for adolescent user satisfaction found by others,^5^ is not replicated in this study.

Similar to most studies,^5^, ^31^, ^51^ both groups generally reported high levels of satisfaction with CAMHS, with parents being significantly more satisfied than adolescents. Nevertheless, compared to previous findings,^3^, ^5^, ^49^ only a modest proportion (6%–8%) of the variance in predictors of parent user satisfaction was explained by the models. On the other hand, both background, clinical and service characteristics were significant predictors for parent user satisfaction. Therefore, these findings highlight the importance of considering various contextual factors when understanding parent satisfaction in CAMHS. Noteworthy, the key background variable of low levels of family stress predicting high levels of parental user satisfaction is supported by others.^3^, ^16^, ^49^ It is likely that parents experiencing high levels of family stress can feel overwhelmed and be less able to advocate their child/adolescents needs, hence communication with the health carer at CAMHS can be deranged leading to lower levels of satisfaction. Therefore, as suggested by Acri et al.,^16^ emphasis on parents' emotional and practical needs might be valuable to enhance collaborative practice. Reduction of stressors may allow parents to be more involved and supportive during treatment. In line with preliminary findings^10^, ^13^ our results also revealed that parents of boys were more likely to be satisfied with the environmental side of the service, like physical sorroundings, timeliness of appointments and access. Parallell to others^10^ we have no theory to explain these results, but a possible explanation of these results may be that the symptoms or presentations of boys' problems are better understood or treated at CAMHS.

Previous literature has proved mixed findings regarding clinical characteristics and satisfaction, from reports of no relationship between clinical characteristics and satisfaction,^15^ to reports of severity or externalizing problems affecting satisfaction.^14^, ^21^, ^22^ Our results contradict previous findings suggesting parents of children with externalizing symptoms are least likely to be satisfied.^14^, ^21^, ^22^ The only significant clinical predictor for parent general satisfaction and satisfaction with care was higher levels of hyperactivity symptoms at intake. A reasonable explanation for this finding could be parents of children/adolescents with higher levels of hyperactive symptoms find CAMHS the right place to get help. Parents may have had a hard time managing the hyperactivity symptoms, and accessing specialized services like CAMHS can provide them with the support and resources they need. As a consequence, they may have felt understood and helped their satisfaction is likely to increase. In addition, recent evidence^9^ suggests parents are least likely to be satisfied if their child/adolescent do not receive a diagnosis at CAMHS. Our results hint at a similar pattern, given that the elevated levels of symptoms at intake increase the likelihood of a diagnosis being confirmed.

Finally, regarding service variables associated with user satisfaction, we found parents who had longer travelling distances to CAMHS were more likely to be satisfied with CAMHS. These results contradict previous research from both Norway and Ireland, showing that having easy access and living close to the service predicted satisfaction.^9^, ^13^ The results may seem counterintuitive, and although the cause for these results is unknown, a likely interpretation may be that parents from rural areas are less likely to previously have sought help. These results suggest that effort should be made to ensure the accessibility for mental health services for all families, regardless of their geographic location. Providing accommodation for parents from rural areas, such as flexible appointment scheduling, intensive treatment options or video consultations, may improve their satisfaction and overall access to care. We cannot rule out that clinicians in the current study, to a greater extent, already accommodate appointments for parents from rural areas. In such a scenario, higher user satisfaction is more likely, according to results by Kapp et al.,^5^ who found satisfaction was higher when they got to be involved in decisions about the frequency of appointments.

### 4.1 Strengths and limitations

This study used routinely collected data from a naturalistic outpatient setting. Encompassing over 200 adolescents and nearly 500 parents, the sample size in the current study is larger than most comparable studies.^3^, ^9^, ^19^, ^52^ As data were collected in ordinary clinical practice, no exclusion criteria were set, except for children <11 years and families self‐excluding by not answering the ESQ. The limitations of using routinely collected data are well‐known and always solicit caution when interpreting results.^53^ By analysing, reporting, and handling missingness by multiple imputations, the current study supplements the extant literature in the field. Regarding possible bias, the acceptable power, and representativeness compared to the total registry sample, especially for parents, advocate findings are generalizable outside the single service studied. Additionally, collecting data over a considerable time period minimizes the likelihood of bias associated with staff. Next, building on the previously rigorously tested ESQ, which has proven strong psychometric characteristics over time for CAMHS strengthens the relevance for CAMHS, both nationally and internationally.

In addition to the mentioned limitations of analysing routinely collected data, it is important to consider the following limitations of the current study in terms of its generalizability. The applicability of the results is restricted by the age range included in the study (adolescents and parents) and the modality of service delivery (outpatient). More specifically, the findings may not accurately represent the user satisfaction of younger children visiting CAMHS. Future research should focus on including younger children to gain a comprehensive understanding of user satisfaction in CAMHS across age ranges. This would require ensuring developmentally appropriate measures and data collection methods. In addition, as data was obtained solely from an outpatient setting, the results may not fully be valid for inpatient populations where care implies a range of different experiences compared to outpatient care. To include the diversity of treatment settings in CAMHS, future research on user satisfaction from inpatient care is needed. Similarly, the lack of information regarding the ethnicity or geographical origin of the family suggests this study cannot account for the potential impact of this variable on user satisfaction. Future research should aim to collect this information to determine the generalizability of the findings across diverse populations. Finally, even though broadly including potential predictors in this study, central variables might have been missed. Specifically, data on service variables like characteristics of interventions might, and adding a measure of the therapeutic alliance would have strengthened the study design and possibly the knowledge of the construct of satisfaction in CAMHS.

### 4.2 Implications

Despite the methodological challenges of measuring satisfaction in CAMHS, this study calls attention to both clinical and research implications. Foremost, the study highlights the need for services to be attentive to collaborative practices that tailor interventions for adolescents and address the emotional and practical needs of parents. Also, considering this study finds adolescents are likely to be more satisfied if symptom levels are low, CAMHS ought to inquire into whether services are designed to fully meet the needs of adolescents with moderate to serious mental health problems. Lastly, addressing disparities in access to care depending on travel distances can contribute to improving user satisfaction.

In terms of future research, it would be useful to extend the current findings by examining younger children and adolescents with experience from inpatient treatment. The inclusion of multiple sites as well as collecting data on ethnicity/geographical origin would also be key for future studies. Furthermore, to reduce the limitations of this and other studies, an experimental design with an even more comprehensive selection of potential predictors would be ideal.

## 5 CONCLUSION

This study revealed predictors of user satisfaction in CAMHS differ for adolescents and parents. For adolescents' higher user satisfaction was associated with good parental mental health and fewer symptoms at intake. Suggesting the importance of addressing parent well‐being at intake in CAMHS interventions. In contrast, parent user satisfaction was predicted by low levels of family stress, higher adolescent hyperactivity symptoms, and longer travelling distances to CAMHS.

These findings emphasize the need for CAMHS to prioritize collaborative practice, attend to the emotional and practical needs of parents, tailor care for adolescents and address accessibility issues for families in rural areas. The study contributes to the existing literature by highlighting specific factors that influence user satisfaction in CAMHS. However, the generality of the current results must be established by future research. In summary, to improve service delivery, CAMHS must emphasize collaborative practice, tailor interventions to symptom severity, address parental needs, and improve accessibility. By implementing these strategies, CAMHS can enhance user satisfaction and ultimately improve outcomes in child and adolescent mental health.

## AUTHOR CONTRIBUTIONS

Yngvild Arnesen and Børge Mathiassen were responsible for the data analysis. Yngvild Arnesen prepared the data set and performed multiple imputations. Yngvild Arnesen wrote the manuscript. Børge Mathiassen and Kjersti R. Lillevoll supervised the writing and commented on the written drafts. All authors have read and approved the final manuscript.

## CONFLICT OF INTEREST STATEMENT

The authors declare no conflict of interest.

## Data Availability

The data that support the findings of this study are available from the University Hospital of North Norway. Restrictions apply to the availability of these data, which were used under license for this study. Data are available to the corresponding author (Yngvild Arnesen) with the permission of the University Hospital of North Norway.

## References

[1] Wolpert M , Vostanis P , Martin K , et al. High integrity mental health services for children: focusing on the person, not the problem. BMJ. 2017;357:j1500. 28373178 10.1136/bmj.j1500

[2] Edbrooke‐Childs J , Jacob J , Argent R , Patalay P , Deighton J , Wolpert M . The relationship between child‐ and parent‐reported shared decision making and child‐, parent‐, and clinician‐reported treatment outcome in routinely collected child mental health services data. Clin Child Psychol Psychiatry. 2015;21(2):324‐338. 26104790 10.1177/1359104515591226

[3] Garland AF , Haine RA , Lewczyk Boxmeyer C . Determinates of youth and parent satisfaction in usual care psychotherapy. Eval Program Plann. 2007;30(1):45‐54. 17689312 10.1016/j.evalprogplan.2006.10.003 PMC1849953

[4] Seibel LF , Peth‐Pierce R , Hoagwood KE . Revisiting caregiver satisfaction with children's mental health services in the United States. Int J Ment Health Syst. 2021;15(1):71. 34454565 10.1186/s13033-021-00493-9 PMC8403344

[5] Kapp C , Perlini T , Jeanneret T , et al. Identifying the determinants of perceived quality in outpatient child and adolescent mental health services from the perspectives of parents and patients. Eur Child Adolesc Psychiatry. 2017;26:1269‐1277. 28382545 10.1007/s00787-017-0985-z

[6] Biering P , Jensen VH . The concept of patient satisfaction in adolescent psychiatric care: a qualitative study. J Child Adolesc Psychiatr Nurs. 2010;23(3):143‐150. 20796097 10.1111/j.1744-6171.2010.00236.x

[7] Biering P . Child and adolescent experience of and satisfaction with psychiatric care: a critical review of the research literature. J Psychiatr Ment Health Nurs. 2010;17(1):65‐72. 20100307 10.1111/j.1365-2850.2009.01505.x

[8] Turchik JA , Karpenko V , Ogles BM , Demireva P , Probst DR . Parent and adolescent satisfaction with mental health services: does it relate to youth diagnosis, age, gender, or treatment outcome? Community Ment Health J. 2010;46(3):282‐288. 20135350 10.1007/s10597-010-9293-5

[9] McNicholas F , Reulbach U , Hanrahan SO , Sakar M . Are parents and children satisfied with CAMHS? Ir J Psychol Med. 2016;33(3):143‐149. 30115187 10.1017/ipm.2015.36

[10] Copeland VC , Koeske G , Greeno CG . Child and mother client satisfaction questionnaire scores regarding mental health services: race, age, and gender correlates. Res Soc Work Pract. 2004;14(6):434‐442.

[11] Garland AF , Aarons GA , Saltzman MD , Kruse MI . Correlates of adolescents' satisfaction with mental health services. Ment Health Serv Res. 2000;2(3):127‐139. 11256722 10.1023/a:1010137725958

[12] Shapiro JP , Welker CJ , Jacobson BJ . The youth client satisfaction questionnaire: development, construct validation, and factor structure. J Clin Child Psychol. 1997;26(1):87‐98. 9118179 10.1207/s15374424jccp2601_9

[13] Holmboe O , Iversen HH , Hanssen‐Bauer K . Determinants of parents' experiences with outpatient child and adolescent mental health services. Int J Ment Health Syst. 2011;5(1):22. 21920037 10.1186/1752-4458-5-22 PMC3182991

[14] Bjørngaard JH , Wessel Andersson H , Osborg Ose S , Hanssen‐Bauer K . User satisfaction with child and adolescent mental health services: impact of the service unit level. Soc Psychiatry Psychiatr Epidemiol. 2008;43(8):635‐641. 18427704 10.1007/s00127-008-0347-8

[15] Stüntzner‐Gibson D , Koren PE , Dechillo N . The Youth Satisfaction Questionnaire: what kids think of services. Fam Soc. 1995;76(10):616‐624.

[16] Acri M , Bornheimer LA , Jessell L , Flaherty HB , McKay MM . The impact of caregiver treatment satisfaction upon child and parent outcomes. Child Adolesc Ment Health. 2016;21(4):201‐208. 27833456 10.1111/camh.12165 PMC5099010

[17] Bjertnaes O , Iversen HH , Skudal KE , Ali WA , Hanssen‐Bauer K . Are parents' geographical origin associated with their evaluation of child and adolescent mental health services? Results from a national survey in Norway. Eur Child Adolesc Psychiatry. 2021;30(7):1027‐1035. 32617774 10.1007/s00787-020-01590-9 PMC8295066

[18] Kjærandsen KS , Brøndbo PH , Halvorsen MB . Determinants of caregiver satisfaction with child neurodevelopmental assessment in neuropaediatric clinics. BMC Health Serv Res. 2021;21(1):139. 33579275 10.1186/s12913-021-06153-5 PMC7881610

[19] Solberg C , Larsson B , Jozefiak T . Consumer satisfaction with the child and adolescent mental health service and its association with treatment outcome: a 3‐4‐year follow‐up study. Nord J Psychiatry. 2015;69(3):224‐232. 25377025 10.3109/08039488.2014.971869

[20] Crow H , Gage H , Hampson S , et al. Measurement of satisfaction with health care: implications for practice from a systematic review of the literature. Health Technol Assess. 2002;6(32):1‐244. 10.3310/hta6320 12925269

[21] Barber AJ , Tischler VA , Healy E . Consumer satisfaction and child behaviour problems in child and adolescent mental health services. J Child Health Care. 2006;10(1):9‐21. 16464930 10.1177/1367493506060200

[22] Godley SH , Fiedler EM , Funk RR . Consumer satisfaction of parents and their children with child/adolescent mental health services. Eval Program Plann. 1998;21(1):31‐45.

[23] Urben S , Gloor A , Baier V , et al. Patients' satisfaction with community treatment: a pilot cross‐sectional survey adopting multiple perspectives: community treatment and satisfaction. J Psychiatr Ment Health Nurs. 2015;22(9):680‐687. 26147874 10.1111/jpm.12240

[24] Fuggle P , McHugh A , Gore L , Dixon E , Curran D , Cutinha D . Can we improve service efficiency in CAMHS using the CAPA approach without reducing treatment effectiveness? J Child Health Care. 2016;20(2):195‐204. 25573900 10.1177/1367493514563856

[25] Brannan AM , Sonnichsen SE , Heflinger CA . Measuring satisfaction with children's mental health services: validity and reliability of the satisfaction scales. Eval Program Plann. 1996;19(2):131‐141.

[26] Gallagher J , Schlösser A . Service users' experiences of a brief intervention service for children and adolescents: a service evaluation. Child Care Pract. 2015;21(4):374‐391.

[27] Church H . Child and Adolescent Mental Health Services in Laois/Offaly: a one year perspective of services (July 1, 2008 to June 30, 2009). Ir J Psychol Med. 2012;29(2):107‐112. 30199957 10.1017/S0790966700017390

[28] Chilvers R , Gratton S , Bernard SH . Satisfaction with a child and adolescent mental health services (CAMHS) intellectual disability service. Adv Ment Health Intellect Disabil. 2013;7:49‐58.

[29] Stacey K , Allison S , Dadds V , Roeger L , Wood A , Martin G . The relationship between change and satisfaction: parents' experiences in a child and adolescent mental health service. Aust N Z J Fam Ther. 2002;23(2):79‐89.

[30] Attride‐Stirling J . Development of Methods to Capture Users' Views of Child and Adolescent Mental Heatlht Services in Clinical Governance Reviews Project Evaluation Report. NHS; 2002.

[31] Brown A , Ford T , Deighton J , Wolpert M . Satisfaction in child and adolescent mental health services: translating users' feedback into measurement. Adm Policy Ment Health Ment Health Serv Res. 2014;41(4):434‐446. 10.1007/s10488-012-0433-9 22829193

[32] Wolpert M , Fonagy P , Frederickson N , et al. Review and Recommendations for National Policy for England for the Use of Mental Health Outcome Measures with Children and Young People. Department of Health; 2008.

[33] Wolpert M , Jacob J , Napoleone E , et al. Child‐ and Parent‐Reported Outcomes and Experience from Child and Young People's Mental Health Services 2011–2015. 2016. CAMHS Press.

[34] Goodman R , Ford T , Richards H , Gatward R , Meltzer H . The development and well‐being assessment: description and initial validation of an integrated assessment of child and adolescent psychopathology. J Child Psychol Psychiatry. 2000;41(5):645‐655. 10946756

[35] Goodman R . Psychometric properties of the Strengths and Difficulties Questionnaire. J Am Acad Child Adolesc Psychiatry. 2001;40(11):1337‐1345. 11699809 10.1097/00004583-200111000-00015

[36] Bunge EL , Maglio AL , Musich FM , Savage C . Consumer satisfaction with private child and adolescent mental health services in Buenos Aires. Child Youth Serv Rev. 2014;47:291‐296.

[37] Uher R , Goodman R . The Everyday Feeling Questionnaire: the structure and validation of a measure of general psychological well‐being and distress. Soc Psychiatry Psychiatr Epidemiol. 2010;45(3):413‐423. 19466369 10.1007/s00127-009-0074-9

[38] Kjærandsen KS , Handegård BH , Brøndbo PH , Halvorsen MB . Parental mental health screening in a neuropaediatric sample: psychometric properties of the Everyday Feeling Questionnaire and variables associated with parental mental health. J Appl Res Intellect Disabil. 2021;34(2):648‐658. 33215810 10.1111/jar.12834

[39] Mann J , Henley W , O'Mahen H , Ford T . The reliability and validity of the everyday feelings questionnaire in a clinical population. J Affect Disord. 2013;148(2‐3):406‐410. 22871532 10.1016/j.jad.2012.03.045

[40] Kornør H , Heyerdahl S . Måleegenskaper ved den norske versjonen av Strengths and Difficulties Questionnaire, selvrapport (SDQ‐S). PsykTestBarn. 2013;2:6.

[41] Kornør H , Heyerdahl S . Måleegenskaper ved den norske versjonen av Strengths and Difficulties Questionnaire, foreldrerapport (SDQ‐P). PsykTestBarn. 2017;1:1.

[42] Shaffer D , Gould MS , Brasic J , et al. A Children's Global Assessment Scale (CGAS). Arch Gen Psychiatry. 1983;40(11):1228‐1231. 6639293 10.1001/archpsyc.1983.01790100074010

[43] Jozefiak T , Hanssen‐Bauer K , Bjelland I . Måleegenskaper ved den norske versjonen av Children's Global Assessment Scale (CGAS). PsykTestB *arn*. 2018;1(3):1‐14.

[44] Lundh A , Kowalski J , Sundberg CJ , Gumpert C , Landén M . Children's Global Assessment Scale (CGAS) in a naturalistic clinical setting: inter‐rater reliability and comparison with expert ratings. Psychiatry Res. 2010;177(1‐2):206‐210. 20334931 10.1016/j.psychres.2010.02.006

[45] Hanssen‐Bauer K , Aalen OO , Ruud T , Heyerdahl S . Inter‐rater reliability of clinician‐rated outcome measures in child and adolescent mental health services. Adm Pol Ment Health Ment Health Serv Res. 2007;34(6):504‐512. 10.1007/s10488-007-0134-y 17846880

[46] Gowers SG , Harrington RC , Whitton A , et al. Brief scale for measuring the outcomes of emotional and behavioural disorders in children. Health of the Nation Outcome Scales for children and Adolescents (HoNOSCA). Br J Psychiatry. 1999;174:413‐416. 10616607 10.1192/bjp.174.5.413

[47] Hanssen‐Bauer K , Langsrud Ø , Kvernmo S , Heyerdahl S . Clinician‐rated mental health in outpatient child and adolescent mental health services: associations with parent, teacher and adolescent ratings. Child Adolesc Psychiatry Ment Health. 2010;4(1):29. 21108776 10.1186/1753-2000-4-29 PMC3003627

[48] Richter J , Hanssen‐Bauer K . Måleegenskaper ved den norske versjonen av Health of the Nation Outcome Scales for Children and Adolescents (HoNOSCA). PsykTestBarn. 2012;1:1.

[49] Perreault M , Rousseau M , Provencher H , Roberts S , Milton D . Predictors of caregiver satisfaction with mental health services. Community Ment Health J. 2012;48(2):232‐237. 21559922 10.1007/s10597-011-9403-z

[50] Bøe T , Øverland S , Lundervold AJ , Hysing M . Socioeconomic status and children's mental health: results from the Bergen Child Study. Soc Psychiatry Psychiatr Epidemiol. 2012;47(10):1557‐1566. 22183690 10.1007/s00127-011-0462-9

[51] McGrath J , Cawley B , McTiernan D , et al. Service user satisfaction with care in a specialist service for young people with attention deficit hyperactivity disorder. Ir J Psychol Med. 2022;1‐8. 10.1017/ipm.2022.15 35361298

[52] Aarons GA , Covert J , Skriner LC , et al. The Eye of the Beholder: youths and parents differ on what matters in mental health services. Adm Pol Ment Health Ment Health Serv Res. 2010;37(6):459‐467. 10.1007/s10488-010-0276-1 PMC2977056 20140489

[53] Wolpert M , Rutter H . Using flawed, uncertain, proximate and sparse (FUPS) data in the context of complexity: learning from the case of child mental health. BMC Med. 2018;16(1):82. 29895295 10.1186/s12916-018-1079-6 PMC5998597

